# DNA Barcoding: A Reliable Method for the Identification of Thrips Species (Thysanoptera, Thripidae) Collected on Sticky Traps in Onion Fields

**DOI:** 10.3390/insects11080489

**Published:** 2020-08-01

**Authors:** Rita Marullo, Francesco Mercati, Gregorio Vono

**Affiliations:** 1 Department of Agriculture, Mediterranean University of Reggio Calabria, Località Feo Di Vito, 89060 Reggio Calabria, Italy; gregorio.vono@unirc.it; 2 Institute of Biosciences and Bioresources, National Research Council, 90129 Palermo, Italy; francesco.mercati@ibbr.cnr.it

**Keywords:** pest thrips, sticky traps, species identification, COI sequences, DNA barcoding, haplotype diversity

## Abstract

**Simple Summary:**

Thrips species (Insecta, Thysanoptera) identification using traditional approach is not an easy. In the present study, DNA barcoding was used to support the thrips species characterization of a wide collection sampled in onion fields. Our findings confirmed the selected method as a simple and accurate approach identifying major thrips species, characterizing successfully nearly 86% of the samples collected in nine main species. The results here reported underlined the role of genetic markers as a valuable and useful method for species identification, especially when the morphological approach is unsure or even impossible.

**Abstract:**

Several thrips species (Insecta, Thysanoptera) are globally known as important crop pests and vectors of viral diseases, but their identification is difficult because of their small body size and inconspicuous morphological differences. Sequencing variation in the mitochondrial cytochrome c oxidase I (COI) region has been proven to be useful for the identification of species of many groups of insect pests. Here, DNA barcoding has been used to identify thrips species collected with the use of sticky traps placed in an open onion field. A total of 238 thrips specimens were analyzed, 151 of which could be identified to species and 27 to genera belonging to the family Thripidae. Fifty-one specimens could not be assigned to any genus, with the closest BLAST match in the GenBank queries being below 98%, whilst six specimens were not recognized as Thysanoptera. The results indicate that, although there are a few pest thrips species not yet barcoded, most of the species that may cause damage to crops in Europe are represented in GenBank and other databases, enabling correct identification. Additionally, DNA barcoding can be considered a valuable alternative to the classic morphology method for identification of major thrips species.

## 1. Introduction

The identification of thrips species (Insecta, Thysanoptera) is not an easy task. The phenotypic and genetic variability in the characters employed for species identification may lead to ambiguous results. Furthermore, specialized morphological keys are required for particular life stages. Hence, morphology-based identification of thrips species demands a high level of expertise. The identification of thrips is mainly based on their biology (e.g., developmental stages and host range) and morphology (e.g., number and patterns of hairs on the wings, head and other parts of the exoskeleton, pattern in the exoskeleton cuticle; segmentation of antenna, ovipositor shape) [1,2]. Morphological identification generally requires microscopic examination of slide-mounted specimens and is based on dichotomous keys. Without expertly slide-mounted specimens, the minute structural details used to diagnose thrips species cannot be studied with accuracy [3,4]. In the process of the identification of thrips species using traditional dichotomous keys, a series of questions must be answered in a correct order, and if an early character is not identifiable, this sequential approach can limit and compromise the identification. Moreover, morphological keys usually are restricted to a target group of thrips species, for instance economically important species or species present within a limited geographical area [5,6,7]. In addition, the identification of larval, prepupal and pupal stages is more difficult than that of adults because of the limited number of available morphological keys for these developmental stages [8], and this is even true for some of the pest species.

Sticky traps of different colors, materials and shapes are used as potential detection and monitoring devices in order to collect information about the number of thrips or other pests associated with a crop under greenhouse and field conditions [9], or for example to study the dispersal distance and direction of the thrips movement out of an orchard, or in quarantine service [10,11]. Trap attractiveness and capture rates of thrips vary according to species [12], trap color [13,14], UV remittance and fluorescence [15], trap size and shape [13,16,17]. Generally, blue and white have been considered as the most preferred colors for several species of thrips [10]. The traps should be at a height of 20–60 cm above the base of the plants, because at this height they will catch more thrips than if they are placed at ground level [18]. The use of sticky traps represents a rapid, simple, cost effective tool for monitoring thrips population densities with little effort. Nevertheless, the minute structural details used to diagnose thrips species may be damaged when using a sticky trap. Morphological methods require the examination of various delicate features of the thrips, including, i.e., the distribution and number of setae across the body and along the forewings that could be seriously damaged using a trap. In order to overcome difficulties linked to species discrimination when morphological examination is not possible or compromised, a variety of molecular methods have been employed for identification of thrips species, e.g., real time PCR [19], polymerase chain reaction restriction fragment polymorphism [20], and microarray assays [21], or the use of gene fragments as internal transcribed spacers [20,22] and mitochondrial cytochrome oxidase (mtCOI) [23,24], of which the COI-based DNA barcoding technique proved to be a highly reliable and efficient method to identify problematic species. The mitochondrial COI is an ideal molecular marker for the purpose of species identification in thrips because it shows a generally low level of intra-species variation but at the same time a considerable level differentiation between species. Herbert et al. [25] suggested a 645 bp fragment at the 5′-end of the mitochondrial cytochrome c oxidase I (COI) as an optimal target for DNA-barcoding because of its generally conserved priming sites. Furthermore, the DNA-barcoding procedure is relatively simple, and it enables detection of groups or biotypes within species [23] often referred to as cryptic species [26,27]. This technique has also been used for the identification of invasive species [28,29] and species complexes [30]. The accurate identification of pest and invasive species is critical, both for control and quarantine, as misidentifications may lead to ineffective control measures [31]. DNA barcoding can also play an important role in insect pest management programs, where both selection and timing of the management practices can be affected by the specific biotype concerned [32,33]. In this study, molecular data have been generated and acquired from NCBI (National Center for Biotechnology Information) and BOLD (Barcode of Life Data System) in order to evaluate the use of COI-based DNA barcoding as a method to identify pestiferous thrips species sampled using sticky traps.

## 2. Materials and Methods

### 2.1. Sampling Sites and Thrips Abundance

The sampled onion fields were situated in the Seeland area (46°57′ N, 7°25′ E; 570 m a.s.l.) in the Canton Bern, Switzerland. Ten different onion fields were selected, and six trap locations were marked inside them. Five thrips specimens were selected at random from each trap, so that a sample of 30 individual thrips was collected for each date of sampling. Blue sticky traps, exposed from the end of June 2015 to the end of August 2015, at a height of 55 cm above the base of the plants, were used and replaced weekly. Collected traps were stored in a refrigerator until the analysis was performed. The data on the thrips collected with the traps were used to evaluate the seasonal abundance of each thrips species.

### 2.2. DNA Extraction

DNA was extracted from every single thrips specimen, using the protocol of Kawasaki [34] with minor modifications: each thrips was placed in a 0.5 mL tube with 100 μL of Proteinase K buffer (10 mM Tris HCl, 1 mM EDTA, 0.5% Tween 20, 50 μg/mL Proteinase K, pH 8.0) using a disposable wooden toothpick. The samples were then homogenized with the use of a mixer mill (Retsch MM300, QIAGEN AG, Basel, Switzerland). The tubes were incubated at 95 °C for 20 min and then stored at −20 °C without any further preparation for subsequent PCR amplification. We tested 240 thrips specimens, for two of which no PCR product was obtained, probably due to mismatches at primer sites causing inefficient primer binding, unsuccessful DNA extraction or presence of inhibitory contaminants in the samples. A total of 238 thrips specimens were thus analyzed.

### 2.3. PCR Amplification and DNA Sequencing

Universal primers listed in Table 1 (LCO-1490 + HCO-2198; C1-J-1751 + C1 N2353; LCO-1490 + C1-N-2191) were used in order to amplify the 5′-end portion COI, under the following conditions: 15 min initial denaturation, followed by 40 cycles of 40 s denaturation at 95 °C, 5 s annealing at 45 °C, ramp to 60 °C in 1 min, hold for 5 s, 2 min extension at 72 °C, followed by a final extension of 7 min at 72 °C.

PCR products were cleaned over silica columns (Qiagen) and a sequencing reaction with 20 ng of PCR product was performed using the BigDye Terminator chemistry (Applied Biosystems). The products of the sequencing reaction were run on an ABI3130xl Applied Biosystems automated sequencer. The DNA extracts were sequenced in both directions to assure accuracy of nucleotide assignments.

### 2.4. Data Analysis

The COI gene sequences were assembled and manually corrected using the software MEGA v.7 [35]. All sequences obtained were compared with those on GenBank by using BLAST tool [36] and species were identified using the BOLD system [37]. To investigate the genetic relationship across the collection sampled a phylogenetic tree including all identified haplotypes for each thrips species was developed. Using PartitionFinder ver 2.1.1 [38], the best fitted model was identified and then the cluster analysis was carried out through maximum likelihood (ML) method [39] by using MEGA v.7 [35]. Bootstrap analysis was performed based on 1000 resampling. The COI sequences of *Heliothrips haemorrhoidalis* (MK484663.1) were used as an outgroup and a bootstrap consensus tree was used to highlight the differences among investigated species. Haplotype network was also constructed for COI using haploNet from the R/pegas package [40]. Finally, we estimated the genetic diversity in terms of segregating sites, nucleotide and haplotype diversity along with Tajima’s D, which tests for neutrality and recent population expansion or contraction, using DNAsp [41]. All sequences generated in this project have been deposited in the GenBank-NCBI (SUB7778457; Table S1).

## 3. Results

BOLD delivers a species identification if the query sequence shows less than 3% divergence from a reference sequence [25,37]. Many studies have shown that sequence variation in the COI gene within thrips species is generally less than 2% [26,32,42,43]. For each individual isolated, a 406 bp long fragment belonging to the COI gene was obtained by PCR amplification and sequencing. Among the specimens analyzed, 151 could be identified to the species level based on the 3% species gap argument. Based on the degree of genetic differentiation between genera, a further 27 specimens could be assigned to the genus level (Table 2), and 51 of the remaining specimens to the family Thripidae, i.e., species of this family for which so far, no barcodes have been deposited in GenBank. The genetic differentiation between each of the last nine specimens of the family Thripidae was as large, or larger, than other Thysanopteran families and hence, these specimens could not be assigned to the Thripidae family. They were therefore recorded as “other species” (Table 2). In total, 74.8% of all field collected samples could thus be assigned to the species or the genus level.

### 3.1. DNA Barcode Analysis

**Figure 1:**
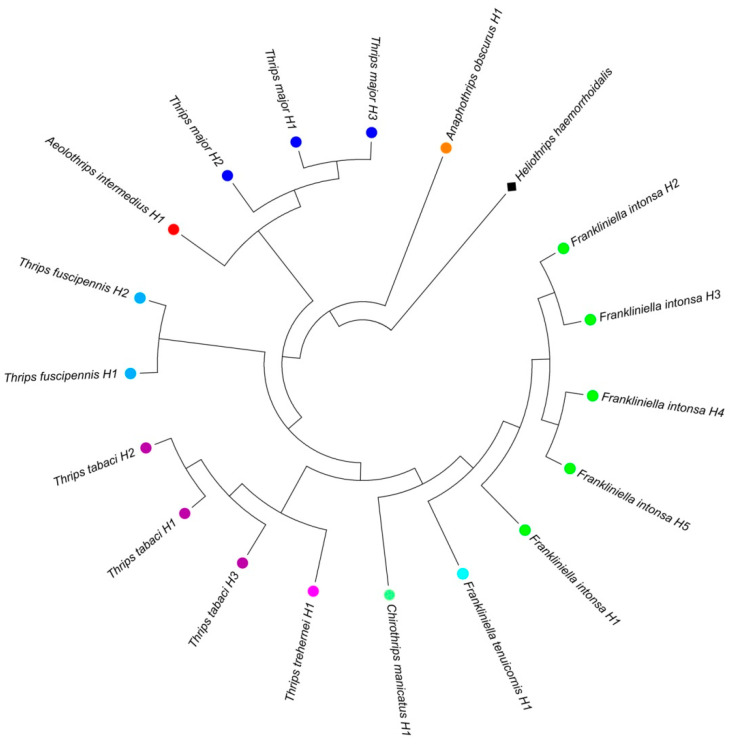
Bootstrap consensus tree generating with maximum likelihood (ML) method and general time reversible (GTR) G + I model showed the genetic relationship among the isolated thrips species obtained by COI sequences. Each species was highlighted with a different color. *Heliothrips haemorrhoidalis* (MK484663.1) was used as outgroup.

**Figure 2:**
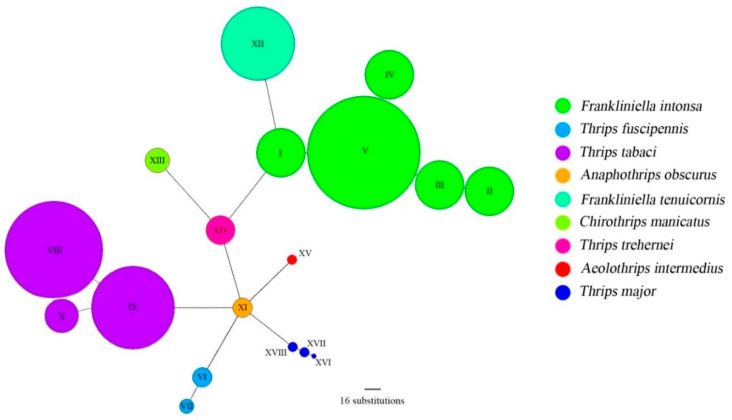
Haplotype networks based on COI sequences. Color codes indicate the different species identified, as shown in the legend, and are the same reported in the phylogenetic analysis (Figure 1). The size of the circles is proportional to the frequencies of the represented haplotypes.

The sequence data analysis was performed through Mega ver.7 software, using the GTR (general time reversible) G + I (Gamma + Invariant) model, suggested by PartitionFinder ver 2.1.1, with ML methodology for the phylogenetic tree construction (Figure 1) and the Tamura-Nei model for genetic distance (Table 3). A total of 151 individual thrips representing nine species were analyzed. In the ML tree only the samples showing different sequences were displayed. The analysis allowed us to separate the species investigated in five main distinct clusters (Figure 1). The two species *F. intonsa* and *F. tenuicornis*, belonging to genus *Frankliniella*, clustered in the same branch together with *C. manicatus*, while *T. trehernei* and *T. tabaci* gathered in another main branch and were separated from *T. fuscipennis*. Finally, the samples belonging to *T. major* were placed in the last cluster, grouping also *Aeolothrips intermedius*, while in the last main branch clustered the samples belonging to *Anaphothrips obscura*. Nearly 65% of branches show a bootstrap value above > 70%, highlighting a significance rate of phylogenetic relationships among the studied species. Thrips individuals were analyzed per species to assess within-species genetic polymorphism (Table 3). Except for five species, all individuals from the same species had identical sequences. A maximum of 17 nucleotide positions were variable in *T. tabaci* and 16 in *T. fuscipennis*. Percent sequence divergence among haplotypes was 2.4% in *T. tabaci* and 2.8% in *T. fuscipennis*. In sharp contrast, differences among haplotypes were only marginal in 63 individuals of *F. intonsa* resulting on an average sequence divergence between haplotypes of 0.5%. The haplotype networks based on COI sequences (Figure 2) showed eighteen different haplotypes belonging to the nine thrips species identified, and both their size (proportional to the frequencies of the relative haplotypes) and distance were in agreement to the phylogenetic analysis.

### 3.2. Seasonal Thrips Abundance on Sticky Traps

**Figure 3:**
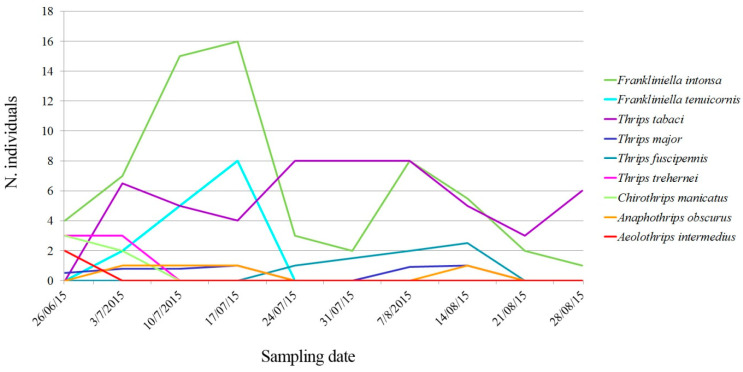
Seasonal fluctuation of thrips species collected on traps. Color codes indicate the different species identified and they are the same reported in the phylogenetic analysis (Figure 1).

Figure 3 shows the seasonal abundance of the common thrips species found on the experimental field. *F. intonsa* was the most common thrips observed in July with the most catches, followed by *T. tabaci*. These two species comprised 42% and 29% of the total thrips complex counted in the field.

## 4. Discussion

In the present study, among the nine thrips species identified, *F. intonsa* and *T. tabaci* were the most abundant due to their pest activity on onion crop, according to literature records for Central Europe [3,22]. Moreover, the presence of *Helianthus* spp. and *Gladiolus* spp. plants, two of the most preferred feeding hosts for both thrips [1], growing around the experimental field could increase the thrips spread to the crop during the flowering time between July and September. The use of DNA barcoding for thrips species identification was first introduced by Brunner [23]. The successful identification of a large proportion of thrips caught on field exposed sticky traps reported here supports the view that COI-based DNA barcoding is a valuable new tool for accurate identification of the agronomically important thrips species. The specific primers we used in the current study enabled successful amplification of most collected specimens which could be, then, properly identified into nine different species through the COI gene fragment-based DNA barcoding. Our results agree with Kadirvel’s [43] suggestions on the identification of thrips species based on the partial COI sequences which were successfully performed for at least 86% of the examined species in their study and reported the method as a simple and accurate means of identifying four major thrips species (*T. palmi*, *T. tabaci*, *S. dorsalis* and *F. occidentalis*). Moreover, the COI sequence was validated to identify and classify thrips species occurring in a crop system where multiple species coexist [43]. This approach is even more appealing with the easy technical cross-transfer among laboratories, enabling the consistency of the process from PCR to the alignment of homologous sequence data and the sharing of sequence data from different and unrelated sources microarray assays [21,31].

In the literature, the use of COI barcoding is reported as an advantage where a target species is characterized by genetic polymorphism [27,32,33,44] and where the accurate and timely identification of a thrips vector is requested, e.g., to understand the epidemiology of arboviruses, and also in quarantine diagnostics [27,45]. Our results have revealed a high genetic variation for the presence of haplotypes referred to in all the nine species tested. According to similar data recorded in the literature for the polymorphism showing up *Frankliniella* species and *Thrips tabaci* group-species [43,46,47], our results need to be improved by using the sequences of conspecific samples collected from different sites. The method is straight-forward, using a single gene region common to all taxa and Sanger-based DNA sequencing under universal conditions. These features allow the standardization of laboratories. The use of reference libraries of barcode sequences for known species is very effective in generating identifications; more than 95% of species in test assemblages of varied animal groups have shown to possess distinctive COI sequences [42]. DNA barcoding has also become an effective tool in revealing cryptic and potentially new species, which has increased the knowledge of biodiversity [30,32,33,46]. Furthermore, it is an important new tool in biosecurity. Many of the monitoring programs to avoid invasion of quarantine pests are challenged by the vast amount of materials at the port of entry, the mostly limited quality as well as the taxonomical breadth of intercepted organisms, often impeding or delaying specimen identification. In addition, morphological identification keys of the majority of agricultural invasive pests, such as thrips, are usually restricted to the adult stage, or the keys for eggs and larvae are often inadequate. COI barcoding-based identification represents a huge advantage by not being limited to any developmental stage, nor the physical integrity of the collected samples. Therefore, the method can be seen as an important new tool for quarantine pests where speed and accuracy of identification are of paramount importance.

## 5. Conclusions

Among the 5800 thrips species worldwide described, only 1% are known as pests causing serious damage or transmitting diseases to growing crops. Consequently, the limited number of thrips [48] in sequence databases has to be taken into account, being referred mainly to the economically important species. Therefore, the ID systems are, in some cases, unable to deliver a species-level identification because many species are not yet associated with barcodes. In this case, the barcoding system can make us confident in yielding a good result for the identification of pest species. Our study has demonstrated that the unidentified specimens through barcodes, will cluster in some genus of family irrelevant to crop protection.

In addition, the results obtained in this study demonstrated some misidentifications which can be attributed to a variety of causes due to technical problems (e.g., ineffective choice of primers). Thus, it is necessary to build robust reference profiles and an agreed framework for the sharing and quality control of the sequence data in order to adopt DNA barcoding on a global scale [31], as well as to allow the classification of crop pest species collected from different vegetables and locations [44]. According to the available literature, the use of genetic markers, such as mtDNA, represents at present, a valuable addition or alternative to the classical methods of species identification [24,33,43,47,49,50,51], especially when the morphological approach is difficult or even impossible.

## Figures and Tables

**Table 1:** List of used primers to amplify the cytochrome c oxidase I (COI) region.

Name	Sequence 5’-3’	Source
HCO-2198	5’ TAA ACT TCA GGG TGA CCA AAA AAT CA 3’	Folmer et al., 1994
LCO-1490	5’ GGT CAA CAA ATC AAA AGA TAT TGG 3’	Folmer et al., 1994
C1-J-1751	5′ GGA TCA CCT CAT ATA GCA TTC CC 3′	Simon et al., 1994
C1-N-2191	5′ CCC GGT AAA AAT TAA AAT ATA AAC TTC 3′	Simon et al., 1994
C1 N2353	5′-GCTCGTG TATCAACGTCTATWCC-3′	Simon et al., 2006

**Table 2:** List of the identified thrips species. Only the samples identified as species (number underlined), for a total of 151 samples, were used in the subsequent analysis.

Genus	Species	No. of Individuals
*Aeolothrips*	*Intermedius*	2
*Anaphothrips*	*Obscura*	4
*Chirothrips*	*Manicatus*	5
*Frankliniella*	other species	2
*Frankliniella*	*Intonsa*	63
*Frankliniella*	*Tenuicornis*	15
*Thrips*	other species	25
*Thrips*	*Fuscipennis*	7
*Thrips*	*Major*	5
*Thrips*	*Tabaci*	44
*Thrips*	*Trehernei*	6
**Total**		**178**

**Table 3:** Genetic diversity and Tajima’s D evaluated for each detected species.

	No. of Sequences	Number of Segregating Sites (S)	Nucleotide Diversity (π)	Standard Deviation of π	Total Number of Haplotypes	Haplotype Diversity (Hd)	Standard Deviation of Hd	Tajima’s D
*A. intermedius*	2	-	-	-	1	-	-	-
*A. obscurus*	4	-	-	-	1	-	-	-
*C. manicatus*	5	-	-	-	1	-	-	-
*F. intonsa*	63	5	0.0032	0.0003	5	0.778	0.027	1.013 (*p* > 0.10)
*F. tenuicornis*	15	-	-	-	1	-	-	-
*T. fuscipennis*	7	16	0.0158	0.0033	2	0.571	0.119	2.220 (*p* < 0.01)
*T. major*	5	5	0.0064	0.0014	3	0.800	0.164	0.562 (*p* > 0.10)
*T. tabaci*	44	18	0.0142	0.0018	3	0.633	0.034	1.670 (*p* > 0.10)
*T. trehernei*	6	-	-	-	1	-	-	-

## Supplementary Materials

The following are available online at https://www.mdpi.com/2075-4450/11/8/489/s1, **Table S1:** List of samples studied in the present work. All samples have been collected on *Allium cepa* as host at Canton Bern (CH; 46°57’ N 7°25’ E). Their accession numbers for the mt-COI sequence are showed.
