# Supplementary material for: DNA Barcoding: A Reliable Method for the Identification of Thrips Species (Thysanoptera, Thripidae) Collected on Sticky Traps in Onion Fields
Source: Insects. 2020 Aug 1;11(8):489. doi: 10.3390/insects11080489 (PMC7469196; doi:10.3390/insects11080489)
Supplement: Supplementary file 1 [file insects-11-00489-s001.pdf]

Table S1. List of samples studied in the present work. All samples have been collected on *Allium cepa* as host at Canton Bern (CH; 46°57’ N 7°25’ E). Their accession numbers for the mt-COI sequence are showed.

Samples	Species	Haplotypes	Accession Number
1	*Frankliniella intonsa*	F.i. – H1	MT782389
2			MT782390
3			MT782391
4			MT782392
5			MT782393
6			MT782394
7			MT782395
8			MT782396
9			MT782397
10			MT782398
11		F.i. – H2	MT782399
12			MT782400
13			MT782401
14			MT782402
15			MT782403
16			MT782404
17			MT782405
18			MT782406
19			MT782407
20			MT782408
21		F.i. – H3	MT782409
22			MT782410
23			MT782411
24			MT782412
25			MT782413
26			MT782414
27			MT782415
28			MT782416
29			MT782417
30			MT782418
31		F.i. – H4	MT782419
32			MT782420
33			MT782421
34			MT782422
35			MT782423
36			MT782424
37			MT782425
38			MT782426
39			MT782427
40			MT782428
41		F.i. – H5	MT782429
42			MT782430
43			MT782431
44			MT782432
45			MT782433
46			MT782434
47			MT782435
48			MT782436

49			MT782437
50			MT782438
51			MT782439
52			MT782440
53			MT782441
54			MT782442
55			MT782443
56			MT782444
57			MT782445
58			MT782446
59			MT782447
60			MT782448
61			MT782449
62			MT782450
63			MT782451
64	*Thrips fuscipennis*	T.f. – H1	MT782452
65			MT782453
66			MT782454
67			MT782455
68		T.f. – H2	MT782456
69			MT782457
70			MT782458
71	*Thrips tabaci*	T.t. – H1	MT782459
72			MT782460
73			MT782461
74			MT782462
75			MT782463
76			MT782464
77			MT782465
78			MT782466
79			MT782467
80			MT782468
81			MT782469
82			MT782470
83			MT782471
84			MT782472
85			MT782473
86			MT782474
87			MT782475
88			MT782476
89			MT782477
90			MT782478
91		T.t. – H2	MT782479
92			MT782480
93			MT782481
94			MT782482
95			MT782483
96			MT782484
97			MT782485
98			MT782486
99			MT782487
100			MT782488
101			MT782489

102			MT782490
103			MT782491
104			MT782492
105			MT782493
106			MT782494
107			MT782495
108		T.t. – H3	MT782496
109			MT782497
110			MT782498
111			MT782499
112			MT782500
113			MT782501
114			MT782502
115	*Anaphothrips obscurus*	A.o. – H1	MT782503
116			MT782504
117			MT782505
118			MT782506
119	*Frankliniella tenuicornis*	F.t. – H1	MT782507
120			MT782508
121			MT782509
122			MT782510
123			MT782511
124			MT782512
125			MT782513
126			MT782514
127			MT782515
128			MT782516
129			MT782517
130			MT782518
131			MT782519
132			MT782520
133			MT782521
134	*Chirothrips manicatus*	C.m. – H1	MT782522
135			MT782523
136			MT782524
137			MT782525
138			MT782526
139	*Thrips trehernei*	T.tr. – H1	MT782527
140			MT782528
141			MT782529
142			MT782530
143			MT782531
144			MT782532
145	*Aeolothrips intermedius*	A.i. – H1	MT782533
146			MT782534
147	*Thrips major*	T.m. – H1	MT782535
148		T.m. – H2	MT782536
149			MT782537
150		T.m. – H3	MT782538
151			MT782539
